# Spondweni Virus in Field-Caught *Culex quinquefasciatus* Mosquitoes, Haiti, 2016

**DOI:** 10.3201/eid2409.171957

**Published:** 2018-09

**Authors:** Sarah K. White, John A. Lednicky, Bernard A. Okech, J. Glenn Morris, James C. Dunford

**Affiliations:** University of Florida, Gainesville, Florida, USA (S.K. White, J.A. Lednicky, B.A. Okech, J.G. Morris, Jr.);; US Navy and Marine Corps Public Health Center, Portsmouth, Virginia, USA (J.C. Dunford)

**Keywords:** Spondweni virus, Zika virus, flaviviruses, Culex quinquefasciatus, viruses, vector-borne infections, Haiti, mosquitoes

## Abstract

Spondweni virus (SPONV) and Zika virus cause similar diseases in humans. We detected SPONV outside of Africa from a pool of *Culex* mosquitoes collected in Haiti in 2016. This finding raises questions about the role of SPONV as a human pathogen in Haiti and other Caribbean countries.

Spondweni virus (SPONV) and Zika virus are closely related flaviviruses that were first described in Africa in 1952 and 1947, respectively (*1*). Humans infected by these viruses have similar clinical manifestations; asymptomatic infections are common, and illness is generally self-limiting (*1*). In the 6 documented human SPONV infections, fever occurred in all. Other symptoms included headache, nausea, myalgia, conjunctivitis, and arthralgia; only 1 SPONV-infected person had maculopapular and pruritic rash (*1*). The similar clinical presentations for these virus infections and reportedly high serologic cross-reactivity have resulted in frequent misdiagnosis (*1*).

Because of the 2015–2016 epidemic of Zika fever in the Western Hemisphere and the link between microcephaly and Zika virus infection, Zika virus has been studied more comprehensively than SPONV (*1*). SPONV was first isolated from *Mansonia uniformis* mosquitoes during virus surveillance in 1955 in South Africa (*2*). No new reports of SPONV surfaced despite continued mosquito surveillance until 1958, when it was identified in 4 additional mosquito species, including *Aedes circumluteolus*, a tropical sylvatic mosquito found in Africa (*2*). Little is known about possible vertebrate hosts, although SPONV antibodies have been detected in birds, small mammals, and ruminants (*2*). In a recent study by Haddow et al. strains of *Ae. aegypti*, *Ae. albopictus*, and *Culex quinquefasciatus* mosquitoes were not susceptible to SPONV infection (*3*).

We detected SPONV from a pool of 7 mixed-sex *Cx. quinquefasciatus* mosquitoes collected in July 2016 during ongoing arbovirus surveillance in Gressier, Haiti. During May–August 2016, we caught 1,756 mosquitoes using Biogents Sentinel traps (BioQuip Products, Rancho Dominguez, CA, USA) within a 10-mile radius in Gressier, a semirural setting. Trap locations were selected based on environmental considerations, low risk for traps being disturbed, and known human arbovirus-caused illnesses in the area (*4*). Trap bags were transported to a field laboratory in Haiti, where mosquitoes were frozen at –20°C, then identified by species and sexed by trained technicians using morphologic keys and identification guides (*5*,*6*). After identification, the mosquitoes were pooled by location, collection date, species (*Ae. aegypti*, *Ae. albopictus*, *Cx. quinquefasciatus*, and other), and sex. All pools were screened for chikungunya virus, dengue virus (DENV) serotypes 1–4, and Zika virus RNA by real-time reverse transcription PCR (rRT-PCR) (Technical Appendix Table 1), as we previously have done with human specimens from Haiti (*4*). Mosquito homogenates positive by rRT-PCR were used for sequencing using primer walking and Sanger sequencing methods as previously reported (*4*; Technical Appendix Table 2). In addition, we confirmed *Aedes* and *Culex* mosquito species by molecular methods (*7*,*8*). In initial screens of a pool of 7 mixed-sex *Cx. quinquefasciatus* mosquitoes (non–blood-fed) collected on July 4, 2016, rRT-PCR results suggested the presence of Zika virus RNA (cycle threshold value 39), but this same pool was negative for chikungunya virus and DENV RNA by rRT-PCR. After unsuccessful attempts to amplify Zika virus–specific amplicons using previously described Zika virus sequencing primers, we used an unbiased sequencing approach after treatment of virions in mosquito homogenate with cyanase (*4*). Because we suspected a closely related virus, we next tested random hexamers and SPONV-specific primers (Technical Appendix Table 3), which resulted in formation of virus-specific amplicons (Technical Appendix). Thereafter, using SPONV primers, we determined a 10,290-nt nearly complete genome and deposited it in GenBank (accession no. MG182017).

The SPONV genome from Haiti shared 10,174 (98.8%) of 10,290 nt identity with a SPONV isolate from mosquitoes in South Africa in 1954 (GenBank accession no. DQ859064) and 9,958 (96.8%) of 10,287 nt identity with the SPONV Chuku strain from blood of a febrile human patient in Nigeria in 1952 (accession no. KX227369) (Table). When compared with the Zika virus reference strain from Uganda (accession no. KY989511), a strain from Puerto Rico (accession no. KU501215), and a strain from Haiti in 2016 (accession no. MF384325), Zika virus and SPONV clearly continue to diverge because the nucleotide and amino acid identities of SPONV are less similar to more recent strains of Zika virus (Table). Few SPONV sequences have been deposited into GenBank, resulting in insufficient information to predict how and when SPONV was introduced in Haiti.

In the Americas and the Caribbean, SPONV is a potential emergent arbovirus and public health threat that manifests clinically with symptoms and signs similar to those of Zika virus infection (*2*,*9*). Misdiagnosis has been documented, and it is possible that SPONV has caused human infection in Haiti but has been misidentified as infection from DENV or other arboviruses (*9*). Little is known about SPONV pathogenesis, host range, and vector competency, especially with vectors present in the Western Hemisphere. Our detection of SPONV in *Cx. quinquefasciatus* mosquitoes raises questions about the role of this species as a vector for this virus and highlights the need for ongoing surveillance for SPONV infection among humans in the Caribbean, combined with studies of potential vector populations.

**Table 1:** Comparison of nucleotide identity of representative strains of SPONV and Zika virus, Haiti*

Virus type and nucleotide GenBank accession no. (country of origin, year)	Nucleotide identity, %						
	SPONV, GenBank accession no.				Zika virus, GenBank accession no.		
	MG182017	DQ859064	KX227369		KY989511	KU501215	MF384325
SPONV MG182017 (Haiti, 2016)	100	98.8	96.8		70.7	70.4	70.4
SPONV DQ859064 (S. Africa, 1954)		100	97.8		70.9	70.6	70.7
SPONV KX227369 (Nigeria, 1952)			100		71.1	70.8	70.8
Zika virus KY989511 (Uganda, 1947)					100	89.0	89.0
Zika virus KU501215 (Puerto Rico, 2015)						100	99.6
Zika virus MF384325 (Haiti, 2016)							100

*SPONV, Spondweni virus.

**Table 2:** Comparison of amino acid identity of representative strains of SPONV and Zika virus, Haiti*

Virus type and protein GenBank accession no. (country of origin, year)	Amino acid identity, %						
	SPONV, GenBank accession no.				Zika virus, GenBank accession no.		
	AVD68687	ABI54480	AOZ57820		ARM59240	AMC13911	ASF57880
SPONV AVD68687 (Haiti, 2016)	100	98.8	98.3		74.1	74.0	74.1
SPONV ABI54480 (S. Africa, 1954)		100	99.1		74.9	74.7	74.8
SPONV AOZ57820 (Nigeria, 1952)			100		74.9	74.8	74.9
Zika virus ARM59240 (Uganda, 1947)					100	96.9	96.9
Zika virus AMC13911 (Puerto Rico, 2015)						100	99.8
Zika virus ASF57880 (Haiti, 2016)							100

*SPONV, Spondweni virus.

Technical Appendix Additional methods for study of Spondweni virus in *Culex quinquefasciatus* mosquitoes, Haiti, 2016.
