# Supplementary material for: Spondweni Virus in Field-Caught Culex quinquefasciatus Mosquitoes, Haiti, 2016
Source: Emerg Infect Dis. 2018 Sep;24(9):1765–7. doi: 10.3201/eid2409.171957 (PMC6106418; doi:10.3201/eid2409.171957)
Supplement: Technical Appendix — Additional methods for study of Spondweni virus in Culex quinquefasciatus mosquitoes, Haiti, 2016. [file 17-1957-Techapp-s1.pdf]

Article DOI: https://doi.org/10.3201/eid2409.171957

Spondweni Virus in Field-Caught *Culex quinquefasciatus* Mosquitoes, Haiti, 2016

Technical Appendix

Methods

Identification of Spondweni Virus Genomic RNA in Mosquito Homogenate

Virus identity was first attained using an unbiased sequencing approach following treatment of virions in mosquito homogenate with cyanase as outlined (*1*). Briefly, 50 μL of mosquito homogenate was diluted with phosphate buffered saline to 500 μL and centrifuged twice at 3,000 × g to pellet debris. The cleared sample was concentrated using an Amicon Ultra-15 centrifugal filter unit with an Ultracel-100 membrane with a molecular mass cutoff of 100 kDa (Millipore, Bedford, MA, USA) at 4,000 × g for 20 min to a volume of ≈80 μl. The volume of the centrifuged fluid was then adjusted to 100 μL by addition of phosphate buffered saline and treated with cyanse nuclease (RiboSolutions, Inc., Cedar Creek, TX, USA) to degrade nucleic acids external to that packaged (and thus protected) in virions (*1*), and as a secondary step, rRNA depletion was performed using a GeneRead rRNA Depletion Kit (QIAGEN, Valencia, CA, USA) to remove residual rRNA. Viral genomic RNA (and/or DNA) were subsequently extracted from virions using a QIAamp Viral RNA Mini Kit (QIAGEN). Because it was not known if there was a DNA or RNA virus, both PCR and reverse transcription PCR (RT-PCR) were performed. For RT-PCR, first-strand synthesis was performed using random 6-mers and an Accuscript High Fidelity 1st strand cDNA kit (Agilent Technologies, Santa Clara, CA, USA). Similarly, PCR was performed using random hexamers and One Taq DNA polymerase (New England Biolabs, Ipswich, MA, USA). PCR amplicons were purified (QIAGEN QIAquick PCR purification kit), and A-tailed with Taq DNA polymerase (New England Biolabs) as a precaution since One Taq, which had been used for PCR, is a mixture of a high-fidelity polymerase and Taq, and therefore, not all amplicons may contain 3′ A-tails. The A-tailed PCR amplicons were then TA-cloned using a commercial kit (pCR2.1 vector with 1 shot TOP10 chemically competent

E. coli cells, ThermoFisher Scientific, Waltham, MA, USA; K204001), and the inserts sequenced using Sanger sequencing. Out of a total of 50 plasmids with TA-cloned inserts, 1 (TA clone 39) contained a 286-bp insert with 98% identity (280/286 nt) with *Spondweni virus* (GenBank accession no. NC_029055.1): 286-bp Insert Derived from Spondweni virus in TA Clone 39:

5′-GCATGAAGGTGTGTTCCAAACAATGTGGCACGTCACAAAAGGTTCGGCCCTTC
GCAGTGGTGAG

GGACGCCTAGATCCATACTGGGGAAACGTGAAGCAGGATTTGATCTCTTACT
GCGGACCATGGAAA

CTGGAGGGGAAATGGGACGGCGTGTCGGAAGTCCAACTGATAGCGGTCGCCC
CAGGTGAGCGCGCCAGAAATGTGCAGACAAAACCAGGAGTGTTCAAGACCACTGAT
GGGGAAATCGGGGCCTTGGCCCTGGACTTCCCAGGCGGAAGTTCAGGC-3′

Sequencing Methods

Sequencing of the complete Spondweni virus genome designated *Culex quinquefasciatus*/Haiti-1/2016 was accomplished using a genome walking strategy based on an approach previously outlined (*1*) using the PCR primers described in Technical Appendix Table 3. Briefly, due to the limited amount of sample material, our preferred strategy of amplifying amplicons of size range 750–800 bp for direct sequencing (*1*) was not possible for the entire sequencing effort. Instead, 3 amplicons of that size range were generated using primer pairs F1 and R1, F2 and R2, and F3 and R3, and 1 that was smaller (289 bp) using primer pairs F7 and R7, and these were directly sequenced to attain consensus sequences. The remaining sequences required amplification of larger amplicons (≈3,000 bp) using primer pairs F4 and R4, F5 and R5, and F6 and R6. Amplicons for sequencing were amplified using Accuscript High Fidelity reverse transcription in the presence of SUPERase-In RNase inhibitor (Ambion, Austin, TX, USA), followed by PCR with Q5 high-fidelity DNA polymerase (New England Biolabs) with denaturation steps performed at 98°C. Smaller amplicons (up to ≈800 bp) were thereafter sequenced directly. The longer amplicons were purified, then smaller amplicons within the size range 750–800 prepared from them using the previously mentioned high-fidelity enzymes, and also directly sequenced using a gene walking approach (primers available upon request). To obtain the 5′ and 3′ ends of the viral genome, we used a 5′ and 3′ system for the Rapid Amplification of cDNA Ends (RACE) in accordance with the manufacturer's protocols (Life

Technologies, Carlsbad, CA, USA) and with primers identified in Technical Appendix Table 3. Bariability at the 5′ and 3′ UTRs made sequence determination impossible using a direct sequencing approach. Sequences were assembled with the aid of Sequencher DNA sequence analysis software v2.1 (Gene Codes, Ann Arbor, MI, USA). The GenBank accession number of Spondweni virus Culex quinquefasciatus/Haiti-1/2016 is MG182017.2.

References

1. Lednicky J, Beau De Rochars VM, El Badry M, Loeb J, Telisma T, Chavannes S, et al. Zika virus outbreak in Haiti in 2014: molecular and clinical data. PLoS Negl Trop Dis. 2016;10:e0004687. PubMed http://dx.doi.org/10.1371/journal.pntd.0004687
2. Lanciotti RS, Kosoy OL, Laven JJ, Panella AJ, Velez JO, Lambert AJ, et al. Chikungunya virus in US travelers returning from India, 2006. Emerg Infect Dis. 2007;13:764–7. PubMed http://dx.doi.org/10.3201/eid1305.070015
3. Santiago GA, Vergne E, Quiles Y, Cosme J, Vazquez J, Medina JF, et al. Analytical and clinical performance of the CDC real time RT-PCR assay for detection and typing of dengue virus. PLoS Negl Trop Dis. 2013;7:e2311. Erratum in: PLoS Negl Trop Dis. 2013;7(7). PubMed http://dx.doi.org/10.1371/journal.pntd.0002311
4. Lanciotti RS, Kosoy OL, Laven JJ, Velez JO, Lambert AJ, Johnson AJ, et al. Genetic and serologic properties of Zika virus associated with an epidemic, Yap State, Micronesia, 2007. Emerg Infect Dis. 2008;14:1232–9. PubMed http://dx.doi.org/10.3201/eid1408.080287

Technical Appendix Table 1. Primers and probes used for real-time reverse transcription PCR detection of chikungunya, dengue, and Zika viruses

Protocol reference	Name (fluor)	Sequence
(*2*)	CHIKV_3855F	GAGCATACGGTTACGCAGATAG
	CHIKV3957c1	TACTGGTGATACATGGTGGTTTC
	CHIKV_3957c2	TGCTGGTGACACATGGTGGTTTC
	CHIKV_3886p1	ACGAGTAATCTGCGTACTGGGACGTA
	CHIKV_3886p2	ACGAGTCATCTGCGTATTGGGACGCA
(*3*)	DENV-1F	CAAAAGGAAGTCGYGCAATA
	DENV-1R	CTGAGTGAATTCTCTCTGCTRAAC
	DENV-1Pr (FAM)	CATGTGGYTGGGAGCRCGC
	DENV-2F	CAGGCTATGGCACYGTCACGAT
	DENV-2R	CCATYTGCAGCARCACCATCTC
	DENV-2Pr (HEX)	CTCYCCRAGAACGGGCCTCGACTTCAA
	DENV-3F	GGACTRGACACACGCACCCA
	DENV-3R	CATGTCTCTACCTTCTCGACTTGYCT
	DENV-3Pr (TX Red)	ACCTGGATGTCGGCTGAAGGAGCTTG
	DENV-4F	TTGTCCTAATGATGCTRGTCG
	DENV-4R	TCCACCYGAGACTCCTTCCA
	DENV-4Pr (Cy5)	TYCCTACYCCTACGCATCGCATTCCG
(*4*)	ZIKV_1086F	CCGCTGCCCAACACAAG
	ZIKV_1162R	CCACTAACGTTCTTTTGCAGACAT
	ZIKV_1107FAM (FAM)	AGCCTACCTTGACAAGCAGTCAGACACTCAA

Technical Appendix Table 2. Zika virus sequencing primers*

Primer name	Sequence 5′–3′
ZIKV-F1	ATG AAA AAC CCA AAA AAG AAA TCC
ZIKV-R1	CAA GCG ATG GCA GCT GCT GCT AAC
ZIKV-F2	GAA TAC ACA AAG CAC TTG ATT AGA GTC
ZIKV-R2	GAA CCA CTC CTT GTG AAC CAA CCA GTG
ZIKV-F3	CTT GAT TGT GAA CCG AGG ACA GG
ZIKV-R3	TCC AAA CAA TGA TTT GAA AGC TGC TC
ZIKV-F3A	TGG AAG CCT AGG ACT TGA TTG TGA AC
ZIKV-F4	CTC ATT GGG CAA GGG CAT CCA TC
ZIKV-R4	CCA GTA GCC TAG ATC ACT GTG TAC
ZIKV-F5	GGA ACA GCT GTT AAG GGA AAG GAG
ZIKV-R5	CCA ATT AGC TCT GAA GAT GAA AGA TAC
ZIKV-F6	CAT TCA AAG TCA GAC CAG CGT TGC
ZIKV-R6	GCA CCA CTC CTT TTT CCA GTC TTG A
ZIKV-F7	GCA GCT GGA GCG TGG TAC GTA TAC G
ZIKV-R7	GAG TGG GTG ACA TTG ACT GCT GTT G
ZIKV-F8	GCC CTT AGA GGG CTT CCA GTG CGT TAT ATG
ZIKV-R8	GAG GCC ATC TTG GAG GTA AAT ATT G
ZIKV-F9	CAC ACT GGC TTG AAG CAA GAA TGC T
ZIKV-R9	GCC ATT TGG TTG TCC TGG GGA GAT CTT TG
ZIKV-F10	GGT GGT GCT CAT ACC TGA GCC AG
ZIKV-R10	CCA AGT AAC TTC CCC TAA AAA TGT TAC AC
ZIKV-F11	CTG GAA CTC CTC TAC AGC CAC TTC AC
ZIKV-R11	GTG GTG GAC ACA CTT TTT ATG GTG TTG
ZIKV-F12	CCC GCA ACT CTA CAC ATG AGA TGT AC
ZIKV-R12	CTA GCC ACA TAT ACC AGA TGG CGC
ZIKV-F13	GAA TTT GGA AAG GCC AAG GGC AG
ZIKV-R13	GGT GGC GGC AGG GAA CCA CAA TG
ZIKV-F14	CTC CAT CTC AAG GAC GGG AGG TC
ZIKV-R14	GCG CGT GGG GTT TTT TGA CTC AGT G
ZIKV-F15	CAT GCT GCC TGT GAG CCC CTC AGA GGA C
ZIKV-R15	CCA CTA GTC CCT CTT CTG GAG ATC C
3 UTR F1	CTA CCT ATC CAC CCA AGT TCG CTA C
3 UTR F2	GTG GCG ACC TTC CCC ACC CTT CAA T
ZIKA 5 UTR R1	CAT ATT GAC AAT CCG GAA TCC TCC

*Data from (*1*).

Technical Appendix Table 3. Spondweni virus sequencing primers*

Primer name	Sequence, 5′–3′	Nt position in SPONV reference strain NC_029055.1
5′ RACE roligo	rArGrC rArUrC rGrArG rUrCrG rGrCrC rUrUrG rUrUrG- rGrCrC rUrArC rUrGrG	N/A
5′-RACE-F	TCGTAGCTCAGCCGGAACAACCGGATGACC	N/A
5′-RACE-R	CCACTTTTCCCCATCTGTTGATGAGGCC	217–190
SPONV-F1	ATGAAGAACCCAAAGAAGGCCGGTA	1–25
SPONV-R1	CCACCAACGCATATCCTGGATTGC	778–755
SPONV-F2	CATGCCTCACAAAAGTTGGAGAC	661–683
SPONV-R2	CCAAAGGAGCCAAGTTCCACCTCAAC	1421–1396
SPONV-F3	CACCAACACGACAAGGAGAAC	1339–1359
SPONV-R3	CTCCTCTCATGGTAGCCTC	2122–2104
SPONV-F4	CACAACAAAAATTACCCACC	2046–2065
SPONV-R4	CTGCATGATGGCACTCACGTAGCTTCC	5007–4981
SPONV-F5	GGGGAAATCGGGGCCTTGG	4867–4885
SPONV-R5	CCTCTCACCTCGAGGACTTGC	7883–7863
SPONV-F6	GCAGAGAACCCGCCAGAAGAG	7685–7705
SPONV-R6	GACAGGGGTCTTGTCCTCCATG	10074–10053
SPONV-F7	GGAACAGAGTGTGGATCATAG	10022–10042
SPONV-R7	TTACAACACACCAGCGCTTGGCCC	10290–10267
3′-RACE-F	GGCGAAACTGAAGAATATAGAG	10207–10228
3′-RACE-F (T25)	TTTTTTTTTTTTTTTTTTTTTTTTT	N/A

*N/A, Not applicable; RACE, Rapid Amplification of cDNA Ends.
